# Successful treatment with lithium carbonate for periodic psychosis of adolescence

**DOI:** 10.1002/pcn5.70133

**Published:** 2025-06-17

**Authors:** Rikuto Christopher Shinohara, Keisuke Inoue, Shun Takayanagi, Yoichi Furutaka, Hikari Yamazaki, Shinya Watanabe

**Affiliations:** ^1^ Department of Psychiatry National Hospital Organization Hokkaido Medical Center Sapporo Hokkaido Japan; ^2^ Department of Psychiatry, Graduate School of Medicine Hokkaido University Sapporo Hokkaido Japan; ^3^ Department of Psychiatry Okamoto Hospital Sapporo Hokkaido Japan

**Keywords:** adolescence, lithium, psychosis

## Abstract

**Background:**

Periodic psychosis of adolescence is a rare psychiatric condition observed in adolescent girls, characterized by recurrent episodes of diverse psychiatric symptoms, including behavioral inhibition, excitement, hyperactivity, hallucinations, and delusions. These episodes closely align with the menstrual cycle. Mood stabilizers, such as lithium carbonate, have demonstrated efficacy in managing this condition.

**Case Presentation:**

We report the case of a 14‐year‐old adolescent girl with periodic psychosis, successfully treated with lithium carbonate. Her symptoms first appeared at age 12 as transient episodes of insomnia, anxiety, and depressive mood. By age 14, she developed hallucinations and persecutory delusions, leading to hospitalization. Initial treatment with risperidone and aripiprazole was discontinued due to suspected neuroleptic malignant syndrome. During hospitalization, she experienced three distinct psychiatric cycles, each comprising a 2‐week period of severe excitement, hyperactivity, hallucinations, and disorganized thinking, followed by a sudden decline in activity levels, with anxiety and fear becoming predominant. Despite adequate treatment with quetiapine and olanzapine, episodes continued to recur. Given the strong correlation between her psychiatric symptoms and menstrual cycle, she was diagnosed with periodic psychosis of adolescence. Lithium carbonate was introduced to prevent further cyclical episodes. The patient was discharged on day 153 of hospitalization. Six months after discharge, she had no recurrence of psychiatric symptoms.

**Conclusion:**

This case underscores the potential efficacy of lithium carbonate for periodic psychosis of adolescence and the importance of understanding this rare but distinctive psychiatric condition.

## BACKGROUND

Periodic psychosis of adolescence, a concept proposed by Yamashita, is a rare psychiatric condition primarily affecting adolescent females. It is characterized by recurrent psychiatric episodes that frequently coincide with the menstrual cycle.^1^ The broad spectrum of symptoms poses challenges for differential diagnosis. Yamashita proposed diagnostic criteria (Table 1) and identified three hallmark features of this condition: (1) behavioral inhibition and stupor, (2) excitement and hyperactivity, and (3) hallucinations and delusions.^1^ Regarding pharmacological treatment, while certain mood stabilizers have demonstrated efficacy, antipsychotics are generally considered less effective.^2^ Among mood stabilizers, lithium carbonate is widely recognized as the first‐line treatment and has been reported to be effective for this condition.^3^ Despite being proposed as a distinct clinical entity, periodic psychosis of adolescence remains poorly recognized, often resulting in delays or inadequacies in appropriate treatment.^1^

**Table 1:** Diagnostic criteria.^1^

**A.** The psychosis occurs periodically in adolescent girls. The first onset is usually in their early teens, and rarely at the beginning of their 20s.
**B.** The duration of disturbed phases is between 1 and 3 weeks; as a rule, not more than 1 month.
**C.** The symptoms become distinct in a day or two and also subside quickly, or are replaced by some other symptoms.
**D.** The symptoms appear repeatedly every month or at rather long intervals. The number of phases is from several to more than 10.
**E.** Most phases are sequentially related to menses. In most cases, the symptoms begin to appear between 10 days prior to, or a few days after, the beginning of the menstrual period. Sometimes they may occur regularly in girls before menarche. Even those who exhibit symptoms usually in accordance with menses may sometimes fall into a disturbed phase without correlation to menses.
**F.** At least one of the following three features is present during a disturbed phase:
(1) Behavioral inhabitation that reaches semistupor or stupor
(2) Unprovoked excitement or hyperactivity
(3) Floating, fragmentary, or transient hallucinations and delusions, or those with definite ideas of reference and persecution.
**G.** At least two of the following four features are present during a disturbed phase:
(1) Persistent or fluctuating anxiety, fear, or irritability
(2) Reduced ability of thinking and understanding. (The patient may have difficulty judging, determining, or even doing simple daily routines.)
(3) Impaired recollection of the events in the disturbed phase
(4) Somatic symptoms, such as insomnia, poor appetite, facial flushing or headache, and so forth.
**H.** Once a disturbed phase is over, the patient becomes perfectly healthy without any residual symptoms.
**I.** The long‐term outcome is favorable. The illness seldom recurs after 30 years of age.

Here, we report a case of periodic psychosis of adolescence with recurrent psychotic symptoms closely associated with the menstrual cycle who was successfully treated with lithium carbonate.

## CASE PRESENTATION

The patient was a 14‐year‐old girl with no history of physical illness or family history of psychiatric disorders. Her growth and development were normal, and she experienced menarche at age 11, with regular menstrual cycles thereafter. Academically, she was a diligent student with excellent grades and actively participated in an athletic club. At age 12, 2 years before admission, she first visited a pediatric clinic with complaints of insomnia, anxiety, and difficulty concentrating. She was prescribed hypnotics as needed. At age 13, 1 year before admission, her symptoms progressed to insomnia, anxiety, and depressed mood, accompanied by vague suicidal ideation. She took a 1‐month break from her club activities, which led to a rapid recovery, allowing her to resume school without noticeable abnormalities.

**Figure 1:**
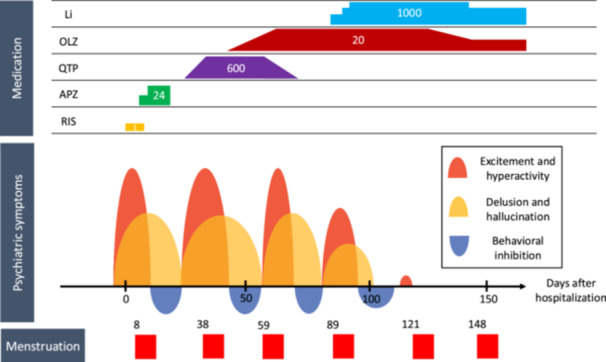
Clinical course of the patient. Initial–maximum doses: aripiprazole (APZ) 12–24 mg/day, lithium carbonate (Li) 400–1000 mg/day, olanzapine (OLZ) 2.5–20 mg/day, quetiapine (QTP) 50–600 mg/day, and risperidone (RIS) 0.5–1 mg/day. The onset of menstrual bleeding in each cycle is also indicated in the figure.

Two weeks before admission, the patient exhibited an elevated mood and hyperactivity, spending an entire week studying without sleep. This was followed by the onset of auditory hallucinations, persecutory delusions, and frequent monologues. As her hyperactivity and excitement intensified, her parents sought medical attention, leading to her admission to a psychiatric hospital. The clinical course and treatment following hospital admission are presented in Figure 1.

Risperidone (1 mg) was initially prescribed; however, due to excessive somnolence, it was replaced with aripiprazole. While her disorganized thinking improved, and she gradually began eating, auditory hallucinations and persecutory delusions persisted. On hospital Day 17, the patient developed fever, tremors, rigidity, and elevated creatine kinase (CK) levels, raising concerns about malignant neuroleptic syndrome. Consequently, all medications were discontinued. On hospital Day 24, she was transferred to the psychiatric department of a general hospital. At the time of transfer, rigidity remained pronounced despite improvement in fever and CK levels, and continued intravenous fluid therapy led to further recovery. Brain magnetic resonance imaging (MRI) and whole‐body computed tomography (CT) revealed no significant abnormalities. Electroencephalography (EEG) revealed no epileptiform discharges or other abnormalities, although the background rhythm was slightly slow at 9–10 Hz. During the interview, she was alert and oriented, though her memory of the prior hospitalization was fragmented. She continued to exhibit persecutory delusions, insisting that her father had admitted her unnecessarily. Quetiapine was initiated and gradually titrated. On hospital Day 27, she exhibited severe excitement, hyperactivity, disorganized thinking, euphoria, and refusal to sleep, leading to seclusion. Quetiapine was increased to 600 mg without sufficient effect, and olanzapine was introduced. Menstrual bleeding occurred on hospital Day 38, after which her excitement, hyperactivity, and disorganized thinking improved. About a week later, her behavior shifted to reduced activity; she spent most of her time in bed, and euphoria was replaced by anxiety, fear, and persecutory delusions, including auditory hallucinations. Menstrual bleeding recurred on hospital Day 59, followed by a return of excitement and hyperactivity. Although the olanzapine dose was increased to 20 mg, her symptoms persisted. After a temporary decline in activity around Day 71, accompanied by anxiety and fear, excitement and hyperactivity resurfaced by Day 85. At this point, the strong correlation between her psychiatric symptoms and menstrual cycle led to a diagnosis of periodic psychosis of adolescence. On hospital Day 87, lithium carbonate was initiated, and olanzapine was gradually tapered. Her levels of excitement and hyperactivity decreased over time, indicating a therapeutic response. Following menstrual bleeding on hospital Day 89, she experienced a brief decline in activity, which gradually resolved, allowing her to adapt well to daily life on the ward. On hospital Day 114, her lithium dose was adjusted to 1000 mg (serum concentration 1.1 mEq/L). On hospital Day 118, she experienced one day of increased talkativeness and elevated mood, but these symptoms did not persist, and no further relapse occurred thereafter. Her lithium dose was reduced to 800 mg (serum concentration 0.6 mEq/L), and she was discharged on hospital Day 153.

After discharge, she returned to junior high school but had difficulty adjusting, eventually transferring to an educational support facility. Six months after discharge, the patient received a regimen of lithium carbonate 800 mg and olanzapine 5 mg. There was no recurrence of the psychiatric symptoms.

## DISCUSSION

The association between psychotic symptoms and the menstrual cycle was first described in the late 19th century by von Kraft‐Ebing, who introduced the concept of “menstrual psychosis.”^4^ Yamashita later defined “periodic psychosis of adolescence” to characterize periodic psychosis occurring during adolescence.^1^ According to Yamashita, this form of psychosis is characterized by episodic disturbances that are closely linked to the menstrual cycle. These episodes often involve alterations in consciousness, ranging from mild to severe, and are typically accompanied by vivid hallucinations and delusions.^1^ They also exhibit significant fluctuations in mood and behavior, which are eventually followed by a gradual and complete return to baseline.^1^ Yamashita proposed the diagnostic criteria for this disorder by identifying three hallmark features: (1) behavioral inhibition or stupor, (2) excitement and hyperactivity, and (3) hallucinations and delusions (Table 1).^1^, ^5^ In the present case, the patient showed a peri‐menstrual cyclical pattern of excitement, hyperactivity, hallucinations, and delusions, followed by behavioral inhibition, closely matching Yamashita's description of periodic psychosis of adolescence.

The understanding of periodic psychosis in adolescence and its inclusion within diagnostic frameworks has evolved over time. In the DSM‐III, “psychotic disorders with atypical presentation” included an example of “transient psychotic episodes associated with the menstrual cycle,” which bears some similarity to periodic psychosis of adolescence.^6^ However, this description was omitted in the DSM‐III‐TR and has remained excluded in subsequent editions.^7^ Likewise, neither the DSM‐5‐TR nor the ICD‐11 includes specific categories that address this group of disorders.^8^, ^9^ The lack of recognition within these diagnostic frameworks has likely contributed to the limited awareness of periodic psychosis of adolescence as a distinct clinical entity. In recent years, menstrual psychosis has been extensively discussed by Brockington.^10^, ^11^ It is defined as an acute onset of psychotic symptoms (such as confusion, delusions, hallucinations, stupor, mutism, or manic states) against a background of normality, with brief duration and full recovery, recurring in rhythm with the menstrual cycle.^11^ When comparing the diagnostic criteria for menstrual psychosis and periodic psychosis of adolescence, the latter appears to represent a narrower clinical entity. For example, menstrual psychosis encompasses various timings within the menstrual cycle and different classifications by stage of reproductive life.^10^ In contrast, periodic psychosis of adolescence is defined in its diagnostic criteria as typically beginning during the premenstrual phase of the menstrual cycle and an onset most commonly occurs during early adolescence, with some cases also appearing in late adolescence. Onset in the early 20s is rare and considered to be the latest possible timing (Table 1).^1^, ^5^ The present case fulfilled the diagnostic criteria for menstrual psychosis; however, it also met the criteria for periodic psychosis of adolescence and closely matched the characteristic symptom patterns proposed by Yamashita. Therefore, we diagnosed and treated this case within the framework of periodic psychosis of adolescence. These conditions are often insufficiently recognized by clinicians due to their low prevalence and limited awareness, and are frequently treated as schizophrenia, schizoaffective disorder, or bipolar disorder. However, given their unique clinical features, it is crucial to make a diagnosis and carefully consider appropriate treatment options.

Pharmacological treatment for periodic psychosis of adolescence has shown limited efficacy with antipsychotics and antidepressants. In contrast, mood stabilizers and hormonal therapies have demonstrated potential benefits.^1^ Among mood stabilizers, carbamazepine and valproate have been reported to be effective.^12^, ^13^ Notably, lithium carbonate has frequently been reported as effective and is considered the first‐line treatment for periodic psychosis of adolescence.^3^, ^5^ Abe reported that lithium successfully prevented episode recurrence in eight out of nine cases, which may be consistent with periodic psychosis of adolescence.^14^ Furthermore, intermittent administration of lithium carbonate starting 1 week before the menstrual period has been shown to prevent a recurrence, suggesting its rapid efficacy and favorable tolerability.^15^ In the present case, prominent psychotic symptoms were initially treated with antipsychotics, but aripiprazole, quetiapine, and olanzapine failed to prevent recurrence. In contrast, lithium carbonate effectively stabilized the patient's condition, highlighting its efficacy in managing periodic psychosis of adolescence. As in the present case, there have been reports of patients whose clinical features were compatible with both menstrual psychosis and periodic psychosis of adolescence, in whom antipsychotics were ineffective while mood stabilizers proved effective.^16^, ^17^ One of these cases initially achieved remission with aripiprazole, but later relapsed and responded well to lithium carbonate.^17^ While in menstrual psychosis, antipsychotics and mood stabilizers are generally considered to be equally positioned treatment options,^10^, ^11^ in periodic psychosis of adolescence, antipsychotics are often ineffective and mood stabilizers are prioritized.^1^ Thus, the difference in treatment approaches between these conditions is noteworthy and may enhance the diagnostic value of periodic psychosis of adolescence.

## CONCLUSION

We report a case of periodic psychosis of adolescence characterized by recurrent psychotic symptoms synchronized with the menstrual cycle, which was successfully treated with lithium carbonate. Clinicians must recognize this disorder and implement the appropriate treatment. Furthermore, lithium carbonate should be prioritized in treating periodic psychosis of adolescence.

## AUTHOR CONTRIBUTIONS

Rikuto Christopher Shinohara conducted the literature review, wrote the manuscript, and revised the draft. Keisuke Inoue contributed to the writing and editing of the manuscript. Shun Takayanagi, Yoichi Furutaka, Hikari Yamazaki, and Shinya Watanabe contributed to editing of the manuscript and participated in the clinical investigations, including patient assessments and data collection. All the authors have read and approved the final version of the manuscript.

## CONFLICT OF INTEREST STATEMENT

The authors declare no conflicts of interest.

## ETHICS APPROVAL STATEMENT

N/A.

## PATIENT CONSENT STATEMENT

Written informed consent for publication of this case report was obtained from the patient and her parents.

## CLINICAL TRIAL REGISTRATION

N/A.

## Data Availability

Data sharing is not applicable to this article, as no datasets were generated or analyzed in the current study.
